# 24-h Urinary Calcium Excretion and Renal Outcomes in Hospitalized Patients with and without Chronic Kidney Disease

**DOI:** 10.3390/jcm12144600

**Published:** 2023-07-11

**Authors:** Xinru Guo, Wanling Wang, Yangyang Ma, Yanjun Liang, Yena Zhou, Guangyan Cai

**Affiliations:** 1 School of Medicine, Nankai University, Tianjin 300071, China; 2 Department of Nephrology, First Medical Center of Chinese PLA General Hospital, Chinese PLA Institute of Nephrology, State Key Laboratory of Kidney Diseases, National Clinical Research Center of Kidney Diseases, Beijing 100853, China; 3 National Engineering Laboratory for Medical Big Data Application Technology, Chinese PLA General Hospital, Beijing 100853, China; 4 Medical Big Data Research Center, Medical Innovation Research Division of Chinese PLA General Hospital, Beijing 100853, China; 5 Department of Information, First Medical Center of Chinese PLA General Hospital, Beijing 100853, China

**Keywords:** chronic kidney disease, prognosis, slope, urinary calcium excretion

## Abstract

This study investigated the impact of 24-h urinary calcium excretion (UCaE) on renal function decline in hospitalized patients with and without chronic kidney disease (CKD). This study enrolled 3815 CKD patients in stages 1–4 and 1133 non-CKD patients admitted to the First Center of the Chinese PLA General Hospital between January 2014 and July 2022. The primary outcome for CKD patients was a composite of CKD progression, defined as a 40% decline in estimated glomerular filtration rate (eGFR) or end-stage kidney disease. Annual eGFR change was the secondary outcome. For non-CKD patients, the primary outcome was an eGFR decline of ≥20% or CKD incidence, while annual eGFR change was the secondary outcome. The association between UCaE and kidney function decline was assessed using Cox proportional hazards and generalized linear models. Primary outcomes were observed in 813 CKD patients and 109 non-CKD patients over a median follow-up of 3.0 and 4.1 years, respectively. For CKD patients, every 1-mmol/d increase in UCaE was associated with a 15% decreased risk of CKD progression. The hazard ratio (HR) was 0.85, with a 95% confidence interval (CI) of 0.77–0.93. For non-CKD patients, the risk of renal function decline decreased by 11%. The multivariate models indicated that there was an annual decrease in eGFR in both CKD and non-CKD patients, with a reduction of 0.122 mL/min/1.73 m^2^/year (*p* < 0.001) and 0.046 mL/min/1.73 m^2^/year (*p* = 0.004), respectively, for every 1-mmol/d increase in UCaE. CKD experiences a decrease in 24-h UCaE as early as stage 1, with a significant decline in stage 4. CKD and non-CKD patients with lower UCaE levels are at an increased risk of renal decline, regardless of other variables.

## 1. Introduction

Maintaining calcium homeostasis is crucial for various physiological processes, including forming cell membranes, exocytosis, enzymatic activity, muscle contraction, neuronal excitability, and bone formation [1,2]. The maintenance of serum calcium levels in the body is mainly regulated by the interplay between intestinal absorption, renal excretion, and bone remodeling. This is achieved through a hormonal feedback loop that involves serum calcium itself and hormones such as parathyroid hormone (PTH), 1,25-dihydroxy vitamin D_3_ (1,25(OH)_2_D_3_), calcitonin, fibroblast growth factor 23 (FGF-23), and alpha-Klotho [3,4]. The kidneys play an important role in regulating calcium balance. Urinary calcium excretion (UCaE), which represents approximately 2% of the glomerular filtered load, indicates the amount of calcium eliminated through urine [5]. On average, healthy individuals excrete around 100 to 200 mg of calcium per 24 h through their kidneys [6]. The majority (85–90%) of calcium ions that are filtered in the kidneys are reabsorbed in the proximal tubule and thick ascending limb through passive paracellular diffusion. The remaining 15% is reabsorbed in the distal convoluted tubule and collecting tubule through a protein called transient receptor potential vanillin member 5 (TRPV5) [7]. Therefore, any issues with the function of the renal tubules can have a significant impact on calcium metabolism.

Epidemiological data have shown that pre-dialysis and dialysis patients with chronic kidney disease (CKD) who have abnormal levels of urinary electrolytes, such as sodium and potassium, are at an increased risk of hypertension, cardiovascular events, and all-cause mortality [8,9]. Previous studies have focused on the relationship between urinary calcium and kidney stone formation or tubular ion channel disease rather than investigating its potential impact on overall kidney damage and function decline [10,11]. There is currently insufficient clinical evidence to support a relationship between 24-h UCaE and the incidence or progression of CKD in populations with or without impaired kidney function.

In this retrospective observational cohort study, we aimed to investigate the association between 24-h urinary calcium levels and risk of kidney function decline in hospitalized patients, regardless of their CKD status. Specifically, we hypothesized that lower and higher urinary calcium levels would increase the risk of declining kidney function. Our analysis was based on the 24-h UCaE results of the participants.

## 2. Methods

### 2.1. Study Design and Population

We established a non-public database of hospitalized patients who underwent 24-h UCaE tests from January 2014 to July 2022 with the assistance of the Department of Information and the Medical Big Data Research Center of the First Medical Center of the PLA General Hospital. The scope of extracting data from large volumes of patient care information includes hospitalization information, nursing records, diagnosis, examination and laboratory records, medical orders, and electronic medical records. Personal information is de-identified to ensure privacy and confidentiality. The study involved screening 20,575 hospitalized patients for the database. Patients under the age of 18, those without serum creatinine, those with unsatisfactory urine volume collection (less than 0.5 L/d or more than 5 L/d) [9], and those with acute kidney injury or renal tubular acidosis or ion disorders were excluded. This resulted in a final sample of 17,836 patients. CKD diagnosis was confirmed using the International Classification of Diseases (ICD-10) as a basis. However, relying solely on ICD coding may not identify all patients with CKD [12]. Therefore, we also utilized CKD criteria, which included laboratory tests and electronic medical records, to identify patients with an eGFR of less than 60 mL/min/1.73 m^2^ or 24-h urine protein levels of 150 mg/day or more for at least 3 consecutive months. For the purpose of our analysis, we defined non-CKD as the absence of a diagnosis of ICD-10 encoding CKD, proteinuria, and impaired kidney function. Our study identified a total of 13,022 patients with CKD, 3958 patients without CKD, and 856 patients with uncertain CKD status. From this group, we enrolled 3815 patients with CKD in stages 1–4 and 1133 patients without CKD who had at least 2 serum creatinine measurements taken more than 1 year apart. We determined eGFR using the chronic kidney disease epidemiology collaboration (CKD-EPI) equation. Figure S1 is the flowchart of the current study.

### 2.2. Exposure and Outcomes

The main focus of the study was on 24-h UCaE, which was measured alongside other 24-h mineral excretion using an automated biochemical analyzer (Cobas c501, Roche, Basel, Switzerland) at the Laboratory Department of the Chinese PLA General Hospital. The reference test range for 24-h UCaE was between 2.5–7.5 mmol/24 h. Baseline information, such as sociodemographic characteristics, medical history, medications, and test results, was collected when the patients were first hospitalized for the UCaE test between 2014 and 2022. Standard assays were used to take all baseline laboratory measurements.

The patients in this study were monitored at the hospital for a minimum of 1 year. In CKD patients, the primary outcome was the progression of CKD, which was defined as a sustained 40% eGFR decline (confirmed after at least 3 months) and/or progression to end-stage renal disease (ESRD) over time. In this study, we defined ESRD as a sustained eGFR of less than 15 mL/min/1.73 m^2^, receipt of long-term maintenance dialysis, or kidney transplant. Based on previous studies and guidelines [13,14], for patients without CKD, the primary outcome was the time it took for their eGFR to decline by 20% or for them to develop incident CKD (defined as eGFR less than 60 mL/min/1.73 m^2^ or 24-h proteinuria greater than or equal to 150 mg/d) that was sustained for more than 3 months. Additionally, we assessed the annual change in eGFR as a secondary outcome. Rapid kidney function decline (RKFD) was defined as a decline in eGFR of more than or equal to 5 mL/min/1.73 m^2^/year [14] for CKD patients and 3 mL/min/1.73 m^2^/year [14] for non-CKD patients. To ensure an accurate determination of ESRD and kidney function decline, we utilized information from follow-up visits to the clinic, ICD codes, medication records, and medical orders. Follow-up time was censored after loss to follow-up, death, meeting non-slope components of the kidney endpoint, or July 2022.

### 2.3. Covariates

Hypertension (HTN), type 2 diabetes mellitus (DM), and cardiovascular disease (CVD) were identified through ICD-10 clinical modification codes or clinical diagnoses recorded in medical records. CVD encompasses a range of conditions, including ischemic heart diseases such as coronary artery disease, angina pectoris, and myocardial infarction, as well as cerebrovascular and peripheral vascular diseases. In order to determine diuretic usage, we considered the use of a diuretic within 3 days prior to the urine electrolyte test, as the effects of a diuretic on calcium excretion can begin within 2–3 days after starting treatment [15]. The causes of CKD were determined through biopsy results and categorized into several groups, including CKD without biopsy, idiopathic IgA nephropathy, idiopathic membranous nephropathy, hypertensive renal damage, diabetic kidney disease, autosomal dominant polycystic kidney disease, and other causes.

### 2.4. Statistical Analysis

In this study, we expressed continuous variables as either mean ± standard deviation (SD) or median [interquartile range (IQR)] and categorical variables as counts with percentages. To account for missing data (missing rate < 6%), we used the random forest-based estimation method, which does not assume normality or require specification of the parametric model. Specifically, we utilized the ‘missForest’ package. To visualize the correlations between baseline 24-h UCaE and kidney function, we employed ‘ggridges’ and ‘ggplot2’.

We used the ‘survminer’ and ‘survival’ R packages to conduct a Kaplan–Meier analysis of UCaE quartiles and kidney outcomes. However, we found that the proportional hazard assumption was not confirmed in CKD patients (global test = 0.0032) when using the ‘cox.zph’ function in the ‘survival’ R package. This assumption was, however, demonstrated in non-CKD patients. To investigate the risk of outcomes based on UCaE in CKD patients, time-dependent Cox regression models were utilized. The multivariate adjustment strategies were designed to account for potential confounding factors between UCaE and decreased renal function based on clinical plausibility. Models of CKD were adjusted for the following baseline covariates: age, sex, current smoking habit, body mass index (BMI), mean arterial pressure (MAP), CKD etiology, DM, CVD, eGFR, 24-h proteinuria, hemoglobin, serum albumin, serum calcium, serum phosphorus, alkaline phosphatase, total cholesterol, triglyceride, urinary sodium excretion, urine potential of hydrogen, angiotensin-converting enzyme (ACE) inhibitor/angiotensin receptor blocker (ARB), glucocorticoids, diuretics, statins, CaCO_3_ supplement, calcium-free phosphorus binders, calcitriol, and vitamin D supplement application. Other vitamin D receptor activators, calcimimetics, and bisphosphonate are rarely used in our medical center, so we did not conduct statistical analysis of these drugs. Non-CKD models were adjusted for age, sex, BMI, MAP, DM, diuretics, serum calcium, eGFR, and 24-h proteinuria. In the current study, serum calcium was found to have a stronger association with UCaE than albumin-corrected calcium. Therefore, serum calcium was used as the co-variable in this study (Table S1).

Linear mixed models were used to estimate the decline slope of eGFR using all eGFR values prior to the initiation of renal replacement therapy. The models included random intercept and random slope for each individual. The generalized linear regression model was utilized, with the annual eGFR change analyzed as either a continuous or binary dependent variable. Follow-up time was stratified to describe the eGFR decline slope in the four UCaE groups. The most recent creatinine recorded minus baseline time was used to calculate follow-up time. *p* was tested using Jonckheere’s trend method.

To test the linearity assumption between UCaE and eGFR decline, we utilized restricted cubic splines through the ‘rms’ package. Specifically, we used 4 knots located at the 5th, 35th, 65th, and 95th percentiles in our models. Additionally, we conducted subgroup analyses and expressed the results through a forest plot using the ‘forestploter’ package. We categorized the patients by age (≥60 or <60 years), sex, BMI (≥25 or <25 kg/m^2^), HTN, DM, and diuretic usage to explore the relationship between UCaE and composite outcomes (primary and secondary outcomes). All statistical tests were 2-sided; a *p*-value < 0.05 was considered statistically significant. R software, version 4.1.3, was used for the analyses (R Project for Statistical Computing).

## 3. Results

### 3.1. Baseline Characteristics

Table S2 displays the median (IQR) age of the study population, indicating that individuals with CKD stages 1–4 had a median age of 47 (35–60), while those without CKD had a median age of 58 (49–67). eGFR was lower in CKD patients (71.1 mL/min/1.73 m^2^) than in non-CKD patients (94.7 mL/min/1.73 m^2^). Additionally, the UCaE was lower in CKD patients (1.7 mmol/d) compared with non-CKD patients (5.0 mmol/d). The median 24-h urine volume was slightly lower in CKD patients (2.0 L/d) compared with non-CKD patients (2.1 L/d). Table 1 displays the baseline characteristics of 3815 patients with CKD stages 1–4 and 1133 without CKD, categorized by UCaE quartiles.

**Figure 1:**
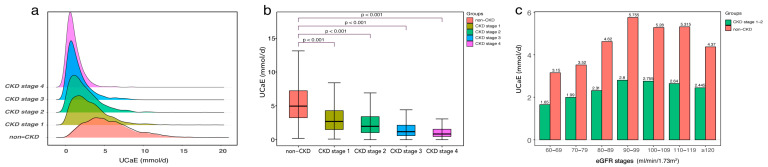
Correlations between 24-h urinary calcium excretion and kidney function. (**a**) A distribution ridge plot of urinary calcium excretion according to the baseline kidney function. (**b**) Comparison of calcium excretion in patients at different stages of CKD and non-CKD. (**c**) Comparison of calcium excretion between 2269 patients with CKD stages 1–2 and 1133 patients without CKD at different eGFR levels. The numbers represent the median value of each group.

The ridge map (Figure 1a) indicates that the 24-h UCaE is more widely distributed and has larger values in non-CKD patients. As the CKD stage increases, the UCaE gap between non-CKD and CKD patients increases significantly (*p* < 0.001, Figure 1b). Figure 1c shows the differences in UCaE between CKD stages 1–2 (n = 2269) and non-CKD (n = 1133) at various eGFR levels. At any level of eGFR, the median UCaE level is consistently higher in non-CKD patients than CKD patients.

### 3.2. Primary Outcomes

Until July 2022, 3815 CKD and 1133 non-CKD patients were followed up for 13,455 and 4856 person-years, respectively. Of the CKD patients, 21.3% (813 individuals) developed the primary outcome, while 9.6% (109 individuals) of non-CKD patients had a sustained change in kidney function.

**Figure 2:**
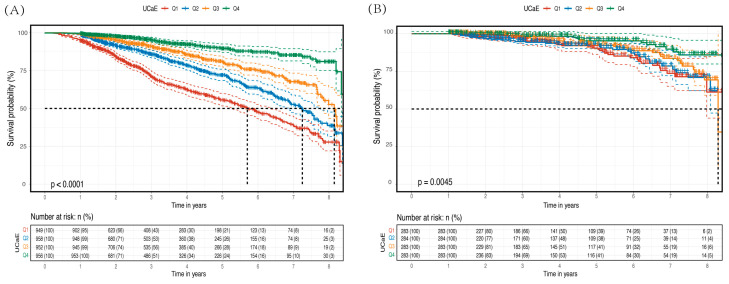
Survival probability and number at risk of renal function decline in strata of 24-h urinary calcium excretion quartiles. (**A**) CKD patients and (**B**) non-CKD patients. Outcomes include time to eGFR decline ≥ 40% or ESRD in CKD and eGFR decline ≥ 20% or incidence of CKD in non-CKD patients.

The Kaplan–Meier analysis indicated that patients with the lowest calcium excretion had a significantly lower probability of not experiencing a decline in renal function compared with those with the highest excretion, regardless of whether they had CKD or not (*p* < 0.0001 and *p* = 0.0045, respectively) (refer to Figure 2). Table 2 presents the results of a time-dependent Cox regression model, which reveals a 15% decrease in the risk of CKD progression for every 1-mmol/d increase in UCaE in CKD patients after full adjustment of the time covariates. The hazard ratio (HR) was 0.85 (95% CI 0.77–0.93), and the *p*-value was less than 0.001. Additionally, patients without CKD exhibited an 11% decreased risk of continued kidney function decline, with an HR of 0.89 (95% CI 0.82–0.95) and a *p*-value of 0.001.

### 3.3. Secondary Outcomes

During a median eGFR follow-up of 3.22 (1.93–5.19) years, the study observed a decline rate of at least 5 mL/min/1.73 m^2^/year in eGFR among 654 CKD patients. In comparison, 102 non-CKD patients had a decline rate of at least 3 mL/min/1.73 m^2^/year over a median follow-up period of 4.05 (2.30–6.22) years. Table 3 shows the correlation between baseline 24-h UCaE and the yearly decline in eGFR in patients. The study found that even after adjusting for variables, there was a noteworthy association between baseline UCaE and the subsequent reduction in renal function in patients with CKD (β = 0.122, *p* < 0.001). The study found that for every 1-mmol/d increase in UCaE, the annual eGFR decline decreased by 0.122 mL/min/1.73 m^2^/year. Additionally, when the dependent variable was binomial, the risk of RKFD (≥5 mL/min/1.73 m^2^/year) was reduced by 10% for each 1-mmol/d increase in UCaE, with an odds ratio of 0.903 (95% CI 0.843–0.969). In non-CKD patients, those with the highest quartile of UCaE had a 60.7% lower risk of rapid eGFR decline than those in the lowest quartile [OR 0.393 (95% CI 0.199–0.775); *p* = 0.007]. For each 1-mmol/d increase in UCaE, the risk of eGFR decline in non-CKD patients decreased by 0.046 mL/min/1.73 m^2^/year.

### 3.4. Further Analysis

**Figure 3:**
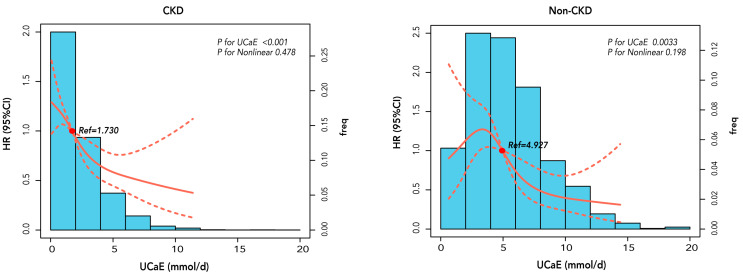
Association between 24-h urinary calcium excretion and risk of renal function decline in 3815 CKD and 1133 non-CKD patients. Data were fit by time-dependent Cox proportional hazards regression models based on restricted cubic splines with four knots and were adjusted for variates. The red dotted lines show 95% confidence intervals. The blue bar charts show the frequency of calcium excretion. Model A was adjusted by age, sex, BMI, MAP, current smoking habit, baseline eGFR, 24-h urine protein, serum calcium, hemoglobin, serum albumin, serum phosphorus, alkaline phosphatase, total cholesterol, triglyceride, urinary sodium excretion, urine potential of hydrogen, CKD etiology, diabetes mellitus, cardiovascular disease, angiotensin-converting enzyme inhibitor (ACE)/angiotensin receptor blocker (ARB) use, glucocorticoids, diuretics, statins, CaCO3 supplements, phosphorus binders, calcitriol, and vitamin D supplements. Model B was adjusted by age, sex, BMI, MAP, eGFR, 24-h urine protein, serum calcium, diabetes, and diuretics use. Abbreviations: CKD, chronic kidney disease; HR (95% CI), hazard ratio (95% confidence interval); UCaE, urinary calcium excretion.

Figure 3 displays the continuous association using restricted cubic splines in models adjusted for variables. The linear hypothesis for predicting CKD progression in CKD was satisfied by UCaE (nonlinear *p* value = 0.478; *p* value for UCaE < 0.001) and non-CKD patients (nonlinear *p* value = 0.198, *p* value for UCaE = 0.0033). When OR was 1, UCaE in CKD and non-CKD patients was 1.73 and 4.93 mmol/d, respectively.

**Figure 4:**
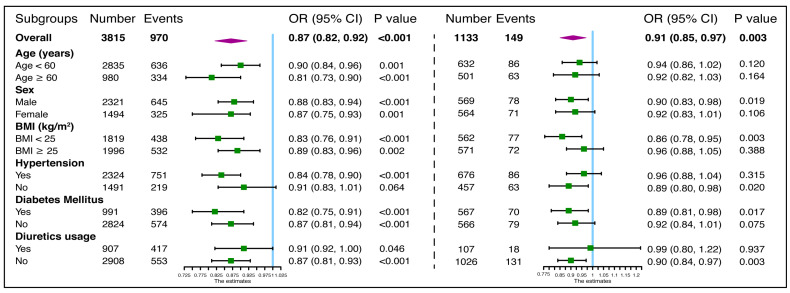
Subgroup analyses of the association between urinary calcium excretion and composite outcomes in patients with and without CKD. Patients were grouped by age, sex, BMI, HTN, DM, and diuretics use. Outcomes are composite outcomes: eGFR decline ≥ 40%, ESRD, and RKFD (slope ≥ 5 mL/min/1.73 m^2^/year) in CKD; incidence of CKD, eGFR decline ≥ 20%, and RKFD (slope ≥ 3 mL/min/1.73 m^2^/year) in non-CKD patients. The odds ratio (OR) was adjusted for age, sex, baseline eGFR, 24-h urine protein, and serum calcium.

In the subgroup analysis of CKD patients, a significant association was found between 24-h UCaE and composite outcomes, except in those with normal blood pressure (*p* = 0.064, Figure 4). Additionally, in non-CKD patients who were male (*p* = 0.019), had a BMI < 25 kg/m^2^ (*p* = 0.003), did not have hypertension (*p* = 0.020), diabetes mellitus (*p* = 0.017), or used diuretics (*p* = 0.003), there was a significant association between UCaE and the decline in kidney function.

Lower UCaE was found to be significantly associated with a faster annual decline in eGFR in patients with CKD, irrespective of the duration of follow-up, as shown in Table 4. In patients without CKD but with a follow-up period of more than five years, a significant association between baseline UCaE and annual eGFR decline was observed (*p* < 0.001).

## 4. Discussion

The study found that the 24-h UCaE in patients with CKD began to decline as early as stage 1 and was severely reduced in stage 4. Patients with early-to-moderate CKD had a UCaE of 1.73 mmol/d, almost one-third of the UCaE in patients without CKD. The study also found that an increase in 24-h UCaE was linked to a lower risk of CKD or CKD progression during the follow-up, regardless of whether the patients had CKD. These associations remained stable even after adjusting for important co-variables for CKD. Sensitivity analyses determined the robustness of these findings. In addition, there was a significant linear trend between UCaE and prognoses. These results suggest that a lower UCaE, rather than a higher one, is a feature and predictor of CKD progression.

The initial theory that reduced UCaE during the progression of kidney disease was due to decreased renal production of 1,25(OH)_2_D_3_ leading to inadequate absorption of calcium from the gastrointestinal tract and increased PTH stimulating enhanced renal tubule calcium reabsorption has been disproven by studies on vitamin D supplementation or decreased PTH, which have failed to alter UCaE in humans [16,17,18]. While hormonal changes may play a role, it is important to note that reduced UCaE in patients with CKD is likely influenced by a combination of factors. These include reduced filtration rate, changes in diet and hormone levels, abnormalities in calcium channels, use of diuretics, and more. In addition to PTH and 1,25(OH)_2_D_3_, recent studies have found that FGF-23 and Klotho are also linked to UCaE. CKD patients with fasting normophosphatemia and normocalcemia have been found to have higher levels of FGF-23 compared to healthy subjects, but a lower fractional excretion of calcium [19]. FGF-23 can activate the enzyme 24,25-hydroxylase (CYP24A1), which degrades vitamin D metabolite 1,25(OH)_2_D_3_. Decreased levels of vitamin D can impair the absorption of calcium in the intestines and its excretion in urine. In addition, PTH, 1,25(OH)_2_D_3_, FGF-23, and Klotho can stimulate TRPV5 activity, leading to increased reabsorption of calcium in the kidneys. This mechanism will be further discussed below.

UCaE is significantly impacted by dietary habits, particularly animal protein, potassium alkali salts, and sodium intake. A clinical study conducted on premenopausal women demonstrated that a decrease in dietary protein intake from 1.1 g/kg to 0.8 g/kg while maintaining similar levels of calcium, phosphorus, and sodium resulted in a 32% reduction in UCaE. However, the levels of serum calcium, PTH, and 1,25(OH)_2_D_3_ remained unchanged [20]. Consuming diets high in meat protein can result in a net acid production, which can lead to chronic metabolic acidosis and renal net acid excretion. This, in turn, can cause hypercalciuria [21,22]. Under the effect of reducing the renal acid load, using alkaline potassium salt can effectively prevent hypercalciuria kidney stones [23]. Patient-oriented and epidemiology studies have demonstrated that a sodium intake of 220 mEq/day or higher is linked to a nearly twofold increase in the likelihood of developing kidney stones, compared to an excretion of less than 120 mEq/day. This is due to the fact that the uptake of calcium in the proximal renal tubular and Henle’s loop is proportional to sodium transport [24]. In normal adults, a decrease of 100 mmol (5.8 g) in sodium salt intake results in a decrease of 1 mmol (40 mg) of calcium [25]. Furthermore, a diet high in sodium intake expands plasma volume and suppresses aldosterone in the renin-angiotensin-aldosterone system, which in turn increases the amount of calcium excreted in urine. Therefore, low protein intake and restriction of sodium or potassium salts in advanced CKD diets may explain the reduced calcium excretion in urine.

Changes in the expression of calcium channels, calcium-binding proteins, calcium pumps, and exchangers in tubules may directly affect UCaE. Calcium reabsorption in the proximal convoluted tubule occurs in parallel with that of sodium and water, mainly through passive paracellular diffusion and solvent drag. The thick ascending limb (TAL) of the loop of Henle are the site of paracellular reabsorption of Ca^2+^ via claudin 16 (CLDN16) and CLDN19, which form heterodimeric paracellular divalent cation channels. The paracellular transport of cations is driven by the lumen-positive voltage generated by Na-K-2Cl cotransporter 2 (NKCC2) activity and luminal K^+^-recycling via the renal outer medullary potassium channel (ROMK) [26]. CLDN14 interacts physically with CLDN16, reducing paracellular permeability by disrupting functional CLDN16/19 heterodimers [26]. Kidney-specific CaSR deletion decreased CLDN14 expression and increased that of the claudin-16 mRNA, reducing the ability of the kidney to excrete calcium [27]. In addition, Toka H et al. [28] also suggested an association of the Claudin14 SNP rs113831133 with lower UCaE. In distal renal tubules, calcium reabsorption is an active transport process involving three steps [29]. The first step requires a calcium influx across the apical membrane, of which TRPV5 has been identified as the responsible protein. The second step is the diffusion of calcium through the cytosol. During this process, calbindin-D28k binds intracellular calcium transported via TRPV5 and shuttles it through the cytosol toward the basolateral membrane, where calcium is extruded via the sodium–calcium exchanger of the plasma membrane calcium-ATPase, which is the final step in this process. Among them, TRPV5 may be the most promising potential target for UCaE regulation. PTH phosphorylates threonine residues in channels and inhibits the endocytosis of caveolae of the channels by activating the cAMP protein kinase A signaling pathway. It increases the opening probability and number of TRPV5 channels on the surface of distal tubule cells by activating protein kinase C pathways, both of which synergistically improve the activity of TRPV5 channels [30]. 1,25(OH)_2_D_3_ enhances the expression of TRPV5 through increased binding of the vitamin D receptor to response elements in the gene promoters to increase respective mRNA concentrations of TRPV5 [31]. Klotho, a protein that exhibits β-glucuronidase activity, tethers TRPV5 on the membrane by binding both TRPV5 and galectin-1, thereby protecting membrane TRPV5 from channel internalization [32]. Studies have also found that FGF-23 [33], sclerostin [34], sex hormones [35,36], lipopolysaccharide [37], urine PH [38], Mucin-1 [39], uromodulin [40], and phosphorylated claudin-16 [41] influence TRPV5 activity.

Thiazide diuretics have been proven to prevent the recurrence of calcium-containing kidney stones. The effects of the diuretics on decreasing calcium excretion can be attributed to a reduced extracellular volume that increases the absorption of urine sodium and water and secondarily increases calcium absorption in the proximal tubule [42]. In addition, thiazide-induced hypocalciuria is also the result of increased calcium reabsorption in the distal tubule and upregulation of TRPV5, calbindins, and other related calcium transport proteins located in the distal tubule [42].

Kidney damage resulting in hypocalciuria has also been noted in animal models [37,43,44]. In partial 5/6 nephrectomy and adenine-enriched dietary intervention to induce experimental CKD animal models, CKD was characterized by enhanced renal expression of the TRPV5, which was a two-fold increase in 5/6 nephrectomy mice than sham-operated mice and a five-fold growth in adenine-treatment mice than in controls. The authors further investigated the stimulation of renal Ca^2+^ processing by inflammatory stimulus and demonstrated elevated TRPV5 mRNA expression after concanavalin administration [43]. Similarly, although an increase in UCaE fraction was observed in animal models with lipopolysaccharide (LPS)-induced acute kidney injury, the total daily UCaE was reduced compared to controls [37]. Endotoxaemia increased renal TRPV5 mRNA abundance 4 h after the injection of LPS and consistently decreased in animals treated for 16 h with LPS. Meanwhile, LPS decreased renal Na^+^/Ca^2+^-exchanger (NCX1) and calbindin-D28K mRNA abundance, causing calcium accumulation in epithelial cells which may lead to a toxic concentration and cause cell damage. Wei Y et al. [44] reported that TRPV5 was upregulated in osteoarthritis articular cartilage, and TRPV5 was potentially mediating Ca^2+^ influx to promote chondrocyte apoptosis in osteoarthritis. However, this apoptosis in tubular cells has yet to be proved. Further research is needed to determine whether increased urinary calcium reabsorption after activation of TRPV5 contributes to renal loss.

Despite remarkable progress in UCaE, the complex mechanism of calcium metabolism and its effects on renal failure remain unclear. Whether reduced UCaE in CKD or CKD higher-risk individuals are in a state of positive calcium balance or reduced net calcium absorption, the mechanism for long-term renal impairment warrants further investigation. There are some limitations to be aware of. First, although the participants in this study broadly represented hospitalized patients with and without CKD regarding age, eGFR, and UCaE, we cannot rule out inherent bias due to the recruitment process, which specifically targeted patients with information on repeated visits. Moreover, the study population may have been selected differently from patients not meeting the inclusion criteria. Second, because PTH, vitamin D, and FGF-23 were not routinely checked in hospitals until the late stages of CKD, we did not have enough information on calcium-regulatory hormones. Despite these, we have adjusted the variables highly correlated with the above hormones, such as serum calcium, phosphorous, and eGFR. In addition, Taylor J et al. [45] reported in 6531 subjects that lower UCaE was associated with a higher risk of developing CKD after adjusting for factors such as PTH and 1,25-dihydroxy vitamin D. Third, we did not investigate the associations between UCaE and mortality due to incomplete death records, so we cannot rule out the competitive risk of death for the progression of CKD. Fourth, the current study was observational, with one calcium excretion test result as the baseline information; thus, this cannot verify the accurate estimation of UCaE and its casual effects on renal outcomes. Fifth, due to the nature of retrospective cohort studies, we lacked data on dietary calcium uptake and fecal excretion. However, this study has various strengths. It is the first study to investigate the association between UCaE and renal outcomes in CKD and non-CKD patients and yields a surprisingly similar trend after adjusting important co-variables in a retrospective cohort study. It also has a relatively large sample size.

In patients with CKD, the 24-h UCaE levels began to decline as early as stage 1 and showed a significant decrease in stage 4 when compared with non-CKD patients. Furthermore, the risk of renal decline was found to be higher in both CKD and non-CKD patients with lower 24-h UCaE levels, regardless of other variables.

## Figures and Tables

**Table 1:** Clinical characteristics of the 4948 hospitalized patients.

	CKD Patients (n = 3815)				non-CKD Patients (n = 1133)			
Quartiles	Q 1	Q 2	Q 3	Q 4	Q 1	Q 2	Q 3	Q 4
24-h UCaE (mmol/d)	~0.84	0.85–1.71	1.72–3.21	3.22~	~3.25	3.26–4.95	4.96–7.23	7.24~
Number	949	958	952	956	283	284	283	283
Basic information								
Age (years)	49 (35, 62)	46 (34, 60)	47 (35, 59)	46 (35, 58)	59 (48, 70)	57 (47, 66)	58 (50, 66)	57 (48, 65)
Male, n (%)	560 (59.0)	573 (59.8)	594 (62.4)	594 (62.1)	138 (48.8)	135 (47.5)	137 (48.4)	159 (56.2)
BMI (kg/m^2^)	24.5 (22.0, 27.0)	25.0 (22.8, 27.7)	25.2 (22.7, 28.1)	25.8 (23.4, 28.4)	24.7 (22.5, 27.3)	25.3 (22.7, 27.6)	24.8 (22.3, 27.2)	25.4 (23.7, 27.8)
MAP (mmHg)	99 (90, 107)	98 (89, 108)	97 (89, 107)	97 (89, 107)	93 (86, 102)	97 (88, 107)	96 (89, 106)	99 (91, 107)
Current smoker, n (%)	99 (10.4)	106 (11.1)	98 (10.3)	125 (13.1)	20 (7.1)	14 (4.9)	18 (6.4)	19 (6.7)
Laboratory test								
eGFR (mL/min/1.73 m^2^)	46.1 (29.6, 72.9)	59.9 (37.9, 89.2)	79.5 (53.4, 100.9)	92.5 (72.0, 107.9)	90.0 (78.3, 103.4)	95.7 (85.7, 105.9)	95.0 (87.0, 103.3)	96.0 (90.1, 106.0)
Hb (g/L)	115 (99, 131)	125 (111, 139)	131 (118, 147)	137 (124, 149)	131.2 ± 16.7	133.9 ± 16.2	134.2 ± 16.8	138.9 ± 15.0
Alb (g/L)	34.3 (26.0, 39.0)	36.6 (30.3, 40.5)	38.0 (31.8, 41.6)	38.6 (32.6, 42.3)	41.2 (38.9, 43.5)	41.6 (39.7, 43.6)	41.9 (39.8, 43.5)	42.1 (40.1, 44.1)
ALP (U/L)	62.0 (50.4, 77.8)	59.6 (49.3, 73.7)	59.4 (48.6, 71.2)	57.0 (46.4, 71.5)	63.9 (51.7, 77.3)	65.2 (52.3, 78.8)	64.5 (54.5, 79.0)	65.9 (55.2, 78.9)
TC (mmol/L)	4.6 (3.8, 5.9)	4.6 (3.9, 5.6)	4.5 (3.8, 5.6)	4.6 (3.8, 5.8)	4.0 (3.4, 4.7)	4.2 (3.6, 4.8)	4.1 (3.5, 4.8)	4.2 (3.5, 4.9)
TG (mmol/L)	1.7 (1.2, 2.6)	1.7 (1.2, 2.5)	1.7 (1.2, 2.5)	1.8 (1.2, 2.6)	1.2 (0.9, 1.8)	1.3 (0.9, 1.9)	1.2 (0.9, 1.7)	1.4 (1.0, 1.9)
24-h proteinuria (g/d)	2.5 (0.9, 4.4)	1.9 (0.8, 4.0)	1.3 (0.5, 3.5)	1.1 (0.4, 2.8)	0.1 (0.0, 0.1)	0.1 (0.0, 0.1)	0.1 (0.0, 0.1)	0.1 (0.0, 0.1)
Serum Ca (mmol/L)	2.1 (2.0, 2.2)	2.2 (2.1, 2.3)	2.2 (2.1, 2.3)	2.2 (2.1, 2.3)	2.2 (2.2, 2.3)	2.3 (2.2, 2.3)	2.3 (2.2, 2.3)	2.3 (2.2, 2.4)
Serum P (mmol/L)	1.2 (1.1, 1.4)	1.2 (1.1, 1.3)	1.2 (1.0, 1.3)	1.2 (1.0, 1.3)	1.1 (1.0, 1.3)	1.1 (1.0, 1.3)	1.1(1.0, 1.3)	1.2 (1.0, 1.3)
24-h UNaE (mmol/d)	94 (58, 138)	118 (79, 163)	130 (89, 184)	166 (116, 224)	114 (78, 160)	134 (94, 179)	156 (120, 208)	195 (142, 247)
24-h UKE (mmol/d)	33.3 (23.9, 43.9)	35.4 (26.7, 46.6)	38.2 (28.8, 49.6)	40.8 (31.3, 54.3)	38.7 (28.8, 51.9)	38.7 (30.4, 50.1)	42.5 (33.3, 52.9)	48.1 (37.5, 62.4)
Comorbidities								
HTN, n (%)	618 (65.1)	618 (64.5)	554 (58.2)	534 (55.9)	160 (56.5)	179 (63.0)	164 (58.0)	173 (61.1)
DM, n (%)	257 (27.1)	214 (22.3)	229 (24.1)	291 (30.4)	120 (42.4)	133 (46.8)	149 (52.7)	166 (58.7)
CVD, n (%)	60 (6.3)	62 (6.5)	57 (6.0)	46 (4.8)	41 (14.5)	24 (8.5)	24 (8.5)	21 (7.4)
Medications								
ACEI/ARB, n (%)	650 (68.5)	683 (71.3)	686 (72.1)	667 (69.8)	104 (36.7)	101 (35.6)	105 (37.1)	108 (38.2)
Diuretics, n (%)	342 (36.0)	223 (23.3)	181 (19.0)	161 (16.8)	39 (13.8)	29 (10.2)	21 (7.4)	17 (6.0)
Glucocorticoids, n (%)	201 (21.2)	195 (20.4)	183 (19.2)	212 (22.2)	10 (3.5)	10 (3.5)	9 (3.2)	5 (1.8)
Statins, n (%)	455 (47.9)	427 (44.6)	428 (45.0)	434 (45.4)	148 (52.3)	126 (44.4)	138 (48.8)	147 (51.9)
CaCO_3_ supplement, n (%)	378 (39.8)	340 (35.5)	330 (34.7)	386 (40.4)	73 (25.8)	82 (28.9)	94 (33.2)	89 (31.4)
Vitamin D_3_ supplement, n (%)	103 (10.9)	84 (8.8)	79 (8.3)	91 (9.5)	40 (14.1)	51 (18.0)	38 (13.4)	53 (18.7)
Calcitriol, n (%)	262 (27.6)	244 (25.5)	244 (25.6)	295 (30.9)	64 (22.6)	62 (21.8)	77 (27.2)	73 (25.8)
P-binders, n (%)	9 (0.9)	2 (0.2)	4 (0.4)	0 (0.0)	0 (0.0)	0 (0.0)	0 (0.0)	0 (0.0)

Notes: P-binders were calcium-free, phosphorus-binding agents. Abbreviations: ACEI/ARB, angiotensin-converting enzyme inhibitor (ACEI)/angiotensin receptor blocker (ARB); Alb, albumin; ALP, alkaline phosphatase; BMI, body mass index; CKD, chronic kidney disease; CVD, cardiovascular disease; DM, diabetes mellitus; eGFR, estimated glomerular filtration rate; G1–G4, stage 1–4; Hb, hemoglobin; HTN, hypertension; MAP, mean arterial pressure; TC, total cholesterol; TG, triglyceride; UCaE, urinary calcium excretion; UNaE, urinary sodium excretion; UKE, urinary potassium excretion.

**Table 2:** Cox proportional hazard model for 24-h UCaE (per 1-mmol/d) and renal outcomes in CKD (3815) and non-CKD patients (1133).

CKD Patients (CKD Progression)			non-CKD Patients (Kidney Function Decline)		
Models	HR (95% CI)	*p*	Models	HR (95% CI)	*p*
Crude	0.64 (0.56, 0.66)	<0.001	Crude	0.89 (0.83, 0.96)	0.001
Model 1 ^a^	0.61 (0.56, 0.66)	<0.001	Model 1 ^d^	0.89 (0.83, 0.95)	<0.001
Model 2 ^b^	0.79 (0.73, 0.86)	<0.001	Model 2 ^e^	0.90 (0.84, 0.96)	0.002
Model 3 ^c^	0.85 (0.77, 0.93)	<0.001	Model 3 ^f^	0.89 (0.82, 0.95)	0.001
No. of events	813		No. of events	109	

Notes: Multivariable model 1 ^a^: age + sex. Multivariable model 2 ^b^: Model 1 ^a^ + baseline eGFR + 24-h urine protein + serum calcium. Multivariable model 3 ^c^: Model 2 ^b^ + CKD causes + BMI + MAP + current smoking + hemoglobin + serum albumin + serum calcium + serum phosphorus + alkaline phosphatase + total cholesterol + triglyceride + urinary sodium excretion + urine potential of hydrogen + diabetes mellitus + cardiovascular disease + angiotensin-converting enzyme inhibitor (ACEI)/angiotensin receptor blocker (ARB) use + glucocorticoids + diuretics use + statins + CaCO_3_ supplements + vitamin D supplements + phosphorus binders + calcitriol. Multivariable model 1 ^d^: age + sex. Multivariable model 2 ^e^: Model 1 ^d^ + baseline eGFR + 24-h urine protein + serum calcium. Multivariable model 3 ^f^: Model 2 ^e^ + BMI + MAP + diabetes mellitus + diuretics use. CKD progression was defined as sustained eGFR decline ≥ 40% or ESRD. Kidney function decline was defined as sustained eGFR decline ≥ 20% or incidence of CKD. Abbreviation: CI, confidence interval; CKD, chronic kidney disease; HR, hazard ratio. UCaE, urinary calcium excretion.

**Table 3:** Association of 24-h urinary calcium excretion with the eGFR annual decline in 3815 CKD and 1133 non-CKD patients.

**UCaE**			**Q 1**	**Q 2**	**Q 3**	**Q 4**	
**CKD Patients (n = 3815)**							
UCaE and annual eGFR decline							
Models	β (95% CI)	*p*		β (95% CI)	β (95% CI)	β (95% CI)	*p* ^#^
Crude	0.168 (0.116, 0.221)	<0.001	Reference	0.639 (0.338, 0.940)	0.878 (0.577, 1.179)	1.110 (0.809, 1.411)	<0.001
Model 1 ^a^	0.165 (0.113, 0.217)	<0.001	Reference	0.596 (0.298, 0.895)	0.854 (0.555, 1.152)	1.062 (0.763, 1.361)	<0.001
Model 2 ^b^	0.136 (0.077, 0.194)	<0.001	Reference	0.564 (0.263, 0.865)	0.765 (0.449, 1.081)	0.944 (0.606, 1.282)	<0.001
Model 3 ^c^	0.122 (0.057, 0.188)	<0.001	Reference	0.420 (0.121, 0.719)	0.583 (0.259, 0.908)	0.810 (0.435, 1.184)	<0.001
UCaE and RKFD							
Models	OR (95% CI)	*p*		OR (95% CI)	OR (95% CI)	OR (95% CI)	*p* ^#^
Crude	0.784 (0.741, 0.830)	<0.001	Reference	0.568 (0.456, 0.708)	0.449 (0.356, 0.565)	0.294 (0.227, 0.380)	<0.001
Model 1 ^a^	0.786 (0.743, 0.832)	<0.001	Reference	0.578 (0.463, 0.722)	0.450 (0.357, 0.569)	0.298 (0.230, 0.385)	<0.001
Model 2 ^b^	0.860 (0.809, 0.914)	<0.001	Reference	0.636 (0.505, 0.801)	0.578 (0.449, 0.744)	0.436 (0.325, 0.584)	<0.001
Model 3 ^c^	0.903 (0.843, 0.969)	0.004	Reference	0.742 (0.578, 0.953)	0.712 (0.538, 0.942)	0.545 (0.387, 0.767)	0.005
Events	654		257	167	136	94	
**non-CKD patients (n = 1133)**							
UCaE and annual eGFR decline							
Models	β (95% CI)	*p*		β (95% CI)	β (95% CI)	β (95% CI)	*p* ^#^
Crude	0.043 (0.016, 0.070)	0.002	Reference	0.018 (−0.213, 0.248)	0.149 (−0.081, 0.379)	0.352 (0.121, 0.582)	0.010
Model 1 ^d^	0.044 (0.017, 0.070)	0.001	Reference	0.041 (−0.187, 0.270)	0.158 (−0.070, 0.386)	0.366 (0.138, 0.595)	0.008
Model 2 ^e^	0.044 (0.017, 0.072)	0.001	Reference	0.020 (−0.207, 0.246)	0.134 (−0.094, 0.362)	0.357 (0.125, 0.590)	0.009
Model 3 ^f^	0.046 (0.019, 0.074)	0.001	Reference	0.032 (−0.195, 0.259)	0.153 (−0.075, 0.382)	0.376 (0.140, 0.612)	0.007
UCaE and RKFD							
Models	OR (95% CI)	*p*		OR (95% CI)	OR (95% CI)	OR (95% CI)	*p* ^#^
Crude	0.897 (0.831, 0.968)	0.005	Reference	0.738 (0.430, 1.266)	0.772 (0.453, 1.318)	0.410 (0.218, 0.771)	0.053
Model 1 ^d^	0.896 (0.830, 0.967)	0.005	Reference	0.714 (0.415, 1.230)	0.773 (0.452, 1.323)	0.401 (0.212, 0.756)	0.045
Model 2 ^e^	0.886 (0.819, 0.958)	0.002	Reference	0.764 (0.439, 1.331)	0.799 (0.460, 1.391)	0.377 (0.195, 0.731)	0.037
Model 3 ^f^	0.889 (0.820, 0.963)	0.004	Reference	0.778 (0.445, 1.361)	0.791 (0.451, 1.388)	0.393 (0.199, 0.775)	0.060
Events	102		34	26	27	15	

Notes: Crude: univariate model. Multivariable model 1 ^a^: age, sex. Multivariable model 2 ^b^: Model 1 ^a^ + baseline eGFR + 24-h urine protein + serum calcium. Multivariable model 3 ^c^: Model 2 ^b^ + CKD causes + BMI + MAP + current smoking + hemoglobin + serum albumin + serum calcium + serum phosphorus + alkaline phosphatase + total cholesterol + triglyceride + urinary sodium excretion + urine potential of hydrogen + diabetes mellitus + cardiovascular disease + angiotensin-converting enzyme inhibitor (ACEI)/angiotensin receptor blocker (ARB) use + glucocorticoids + diuretics use + statins + CaCO_3_ supplements + vitamin D supplements + phosphorus binders + calcitriol. Multivariable model 1 ^d^: age, sex. Multivariable model 2 ^e^: Model 1 ^d^ + eGFR + 24-h urine protein + serum calcium. Multivariable model 3 ^f^: Model 2 ^e^ + MAP + BMI + diabetes mellitus + diuretics use. *p* ^#^, *p* for trend. Abbreviations: CI, confidence interval; CKD, chronic kidney disease; eGFR, estimated glomerular filtration rate; OR, odd ratio; RKFD, rapid kidney function decline; UCaE, urinary calcium excretion. RKFD was defined as eGFR slope ≥ 5 mL/min/1.73 m^2^ and ≥3 mL/min/1.73 m^2^ in CKD and non-CKD patients, respectively.

**Table 4:** eGFR annual decline in participants grouped by years of follow-up and quartiles of 24-h urinary calcium excretion in 3815 CKD and 1133 non-CKD patients.

**eGFR Annual Decline in CKD Participants**						
**Follow-up**	**n**	**Q 1 (n = 949)**	**Q 2 (n = 958)**	**Q 3 (n = 952)**	**Q 4 (n = 956)**	***p*** ^**#**^
Overall	3815	−2.94 (−5.35, −1.20)	−2.33 (−4.10, −0.95)	−1.99 (−3.47, −0.89)	−1.99 (−3.13, −0.85)	<0.001
1–2 years	1747	−2.74 (−5.47, −1.41)	−2.32 (−3.55, −1.31)	−2.21 (−3.27, −1.29)	−2.22 (−3.11, −1.41)	<0.001
3–4 years	1035	−3.40 (−5.80, −1.25)	−2.32 (−4.56, −0.74)	−1.93 (−3.85, −0.68)	−1.67 (−3.49, −0.63)	<0.001
5~ years	1033	−2.65 (−4.93, −0.76)	−2.37 (−4.41, −0.92)	−1.67 (−3.50, −0.65)	−1.40 (−2.91, −0.46)	<0.001
**eGFR Annual Decline in non-CKD Participants**						
**Follow-up**	**n**	**Q 1 (n = 283)**	**Q 2 (n = 284)**	**Q 3 (n = 283)**	**Q 4 (n = 283)**	***p*** ^**#**^
Overall	1133	−1.68 (−2.37, −0.83)	−1.58 (−2.22, −0.84)	−1.43 (−2.21, −0.72)	−1.34 (−2.01, −0.64)	0.002
1–2 years	399	−2.16 (−2.65, −1.45)	−2.13 (−2.49, −1.70)	−2.09 (−2.55, −1.58)	−1.97 (−2.41, −1.41)	0.198
3–4 years	283	−1.37 (−2.18, −0.56)	−1.24 (−1.90, −0.76)	−1.30 (−1.91, −0.73)	−1.55 (−1.95, −1.02)	0.286
5~ years	451	−1.32 (−1.99, −0.77)	−0.91 (−1.62, −0.43)	−0.94 (−1.57, −0.37)	−0.80 (−1.25, −0.24)	<0.001

Notes: The annual decline in eGFR over the UCaE quartile was described by stratification of follow-up time, calculated from the most recent creatinine record time minus baseline. *p* ^#^: *p* for tend of Jonckheere’s test.

## Data Availability

The data that support the findings of this study are available from the corresponding author upon reasonable request.

## Supplementary Materials

The following supporting information can be downloaded at: https://www.mdpi.com/article/10.3390/jcm12144600/s1, Supplemental Figure S1. The flowchart of patient screening. Supplemental Table S1. Spearman analysis (Rho) of the correlation between serum calcium and 24 h urinary calcium excretion before and after albumin adjustment. Supplemental Table S2. The characteristics of the CKD and non-CKD patients.
