# Supplementary material for: 24-h Urinary Calcium Excretion and Renal Outcomes in Hospitalized Patients with and without Chronic Kidney Disease
Source: J Clin Med. 2023 Jul 11;12(14):4600. doi: 10.3390/jcm12144600 (PMC10380443; doi:10.3390/jcm12144600)
Supplement: Supplementary file 1 [file jcm-12-04600-s001.zip › jcm-2428804-supplementary.pdf]

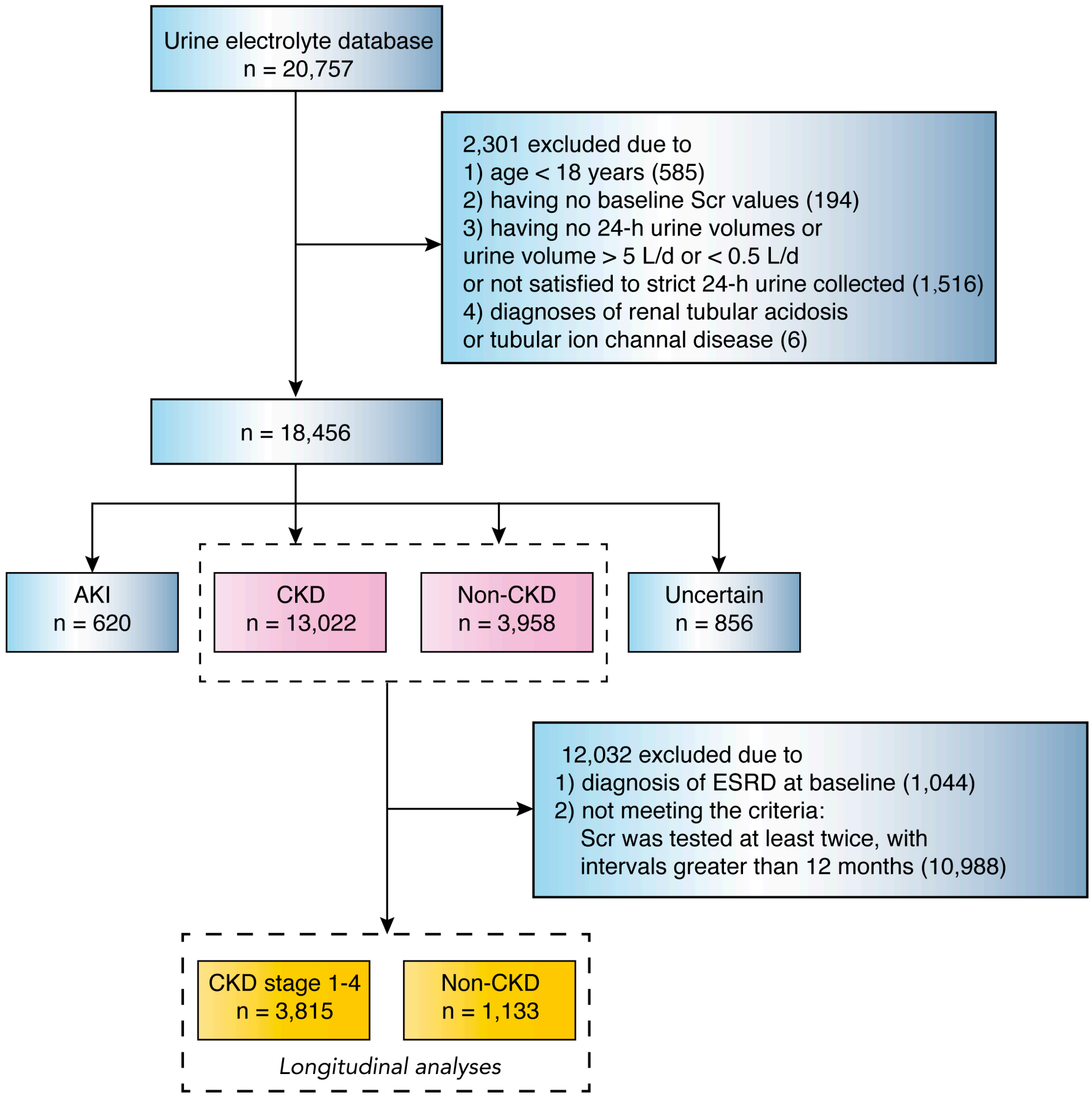

Figure S1. The flowchart of patients screening.

Table S2. The characteristics of the CKD and Non-CKD patients.

Variables	Overall N=4,948	Non-CKD N=1,133	CKD N=3,815	P
Age (year)	50 (37, 62)	58 (49, 67)	47 (35, 60)	<0.001
Female, n (%)	2058 (41.6)	564 (49.8)	1494 (39.2)	<0.001
BMI (kg/m^2)	25.1 (22.7, 27.8)	25.1 (22.8, 27.5)	25.2 (22.7, 27.8)	0.326
MAP (mmHg)	97 (89, 107)	96 (88, 105)	98 (89, 107)	<0.001
eGFR(ml/min/1.73m^2)	81.8 (51.9, 100.7)	94.7 (85.9, 104.4)	71.1 (43.6, 97.5)	<0.001
CKD stages, n (%)				<0.001
G1, n (%)	1258 (25.4)	0 (0.0)	1258 (33.0)	
G2, n (%)	1011 (20.4)	0 (0.0)	1011 (26.5)	
G3, n (%)	1063 (21.5)	0 (0.0)	1063 (27.9)	
G4, n (%)	483 (9.8)	0 (0.0)	483 (12.7)	
24-h UCaE (mmol/d)	2.2 (1.0, 4.2)	5.0 (3.2, 7.2)	1.7 (0.8, 3.2)	<0.001
24-h Urine volume (L/d)	2.0 (1.5, 2.6)	2.1 (1.6, 2.7)	2.0 (1.5, 2.6)	0.005

Note: Continuous variables were expressed as median [interquartile range (IQR)], and categorical variables as counts with percentages. Baseline features were compared by chi-square test and Mann-Whitney U test.

BMI, body mass index; CKD, chronic kidney disease; MAP, mean arterial pressure; eGFR, estimated glomerular filtration rate; G1-G4, stage 1-4**;** UCaE, urinary calcium excretion.

Table S1. Spearman analysis (Rho) of the correlation between serum calcium and 24h urinary calcium excretion before and after albumin adjustment

	Measured [Ca]	P	Corrected [Ca]	P
**UCaE (CKD)**	0.238	<0.001	0.112	<0.001
**UCaE (Non-CKD)**	0.098	0.001	0.067	0.024

Note: The corrected serum calcium is adjusted by albumin, and the formula is:

Corrected [Ca] =Measured total [Ca] + (0.8×(4.0-[Alb])); unit of albumin is g/dl.
